# Efficacy of histology-agnostic and molecularly-driven HER2 inhibitors for refractory cancers

**DOI:** 10.18632/oncotarget.24188

**Published:** 2018-01-12

**Authors:** Luc Cabel, Alina Fuerea, Ludovic Lacroix, Capucine Baldini, Patricia Martin, Antoine Hollebecque, Sophie Postel-Vinay, Andrea Varga, Rastilav Balheda, Anas Gazzah, Jean-Marie Michot, Aurélien Marabelle, Etienne Rouleau, Eric Solary, Thierry De Baere, Eric Angevin, Jean-Pierre Armand, Stefan Michiels, Jean Yves Scoazec, Samy Ammari, Fabrice André, Jean-Charles Soria, Christophe Massard, Loic Verlingue

**Affiliations:** ^1^ Drug Development Department (DITEP), Gustave Roussy Department of Medical Oncology, Faculté de Medicine Paris-Sud XI, Villejuif, France; ^2^ Laboratory of Translational Research and Biological Resource Center, AMMICA, INSERM US23/CNRS UMS3655 Department of Medical Biology and Pathology, Gustave Roussy, Villejuif, France; ^3^ Department of Medical Biology and Pathology, Gustave Roussy, Villejuif, France; ^4^ Gustave Roussy Department of Medical Oncology, Faculté de Medicine Paris-Sud XI, Villejuif, France; ^5^ Inserm Unit UMR 1170, Université Paris Saclay, Université Paris-Sud, Gustave Roussy, Villejuif, France; ^6^ Department of Interventional Radiology, Gustave Roussy, Faculté de Medicine Paris-Sud XI, Villejuif, France; ^7^ Service de Biostatistique et d'Épidémiologie, Gustave Roussy, Villejuif, France; ^8^ Department of Radiology, Gustave Roussy, Faculté de Medicine Paris-Sud XI, Villejuif, France; ^9^ Inserm Unit U981, Université Paris Saclay, Université Paris-Sud, Gustave Roussy, Villejuif, France

**Keywords:** ERBB2/HER2 mutation, ERBB2/HER2 amplification, targeted therapy, trastuzumab, personalized medicine

## Abstract

A targeted therapy is recommended in case of *ERBB2* alteration for breast and gastric carcinomas, but miscellaneous other tumor types are *ERBB2-*altered at low prevalence. Broadening the administration of HER2 inhibitors across tumor types and genomic alterations could benefit to patients with refractory metastatic tumors.

Targeted next-generation-sequencing (tNGS) and comparative genomic hybridization array (CGH) have been performed on fresh tumor biopsies of patients included in the MOSCATO-01 and ongoing MOSCATO-02 trials to administrate HER2 inhibitors in case of *ERBB2* pathogenic mutation of amplification.

Between December 2011 and January 2017 a molecular analysis was performed for 934 patients (759 CGH and 912 tNGS). A novel *ERBB2* alteration has been found in 4.7% (*n =* 44/934), including 1.5% (*n =* 14/912) *ERBB2* mutations, and 4% (*n =* 30/759) *ERBB2* amplifications.

A matched HER2 inhibitor was administrated to 70% (31/44) of patients and consisted in trastuzumab plus chemotherapy for 90% of them (28/31). On the 31 evaluable patients, 1 complete response (CR), 10 partial response (PR) and 2 stable disease (SD) >24 weeks were observed accounting for a clinical benefit rate (CBR) of 42% (*n =* 13/31, 95% CI 25–61%). Besides breast and oesogastric carcinomas, 19 patients affected by 8 different tumor types had a CBR of 25% for *ERBB2* mutations (*n =* 2/8, 95% CI 3%–65%, with 2 PR) and 64% for *ERBB2* amplifications (*n =* 7/11, 95% CI 31%–89%; with 1 CR, 4 PR, 2 SD).

*ERBB2* genomic alterations were diffuse across metastatic tumor types and signs of efficacy emerged for HER2 targeted treatments, especially in case of *ERBB2* amplifications or a p.S310Y *ERBB2* mutation.

## INTRODUCTION

The diagnostic of amplification in the *ERBB2* oncogene leading to the overexpression of the HER2 protein constitutes a paradigm for the use of biomarkers in oncology since trastuzumab, an anti-HER2 antibody, have revolutionized the outcome of *ERBB2*-amplified metastatic breast cancer patients [1]. Routine screening of HER2 overexpression or *ERBB2* amplification is therefore recommended for breast and oesogastric adenocarcinomas on the tumor sample used for the diagnosis [2, 3]. HER2 overexpression or *ERBB2* amplification are observed in approximately 20% of metastatic breast cancers [4] and 20% of metastatic oesogastric adenocarcinomas [5]. In breast cancer, it has been shown that *ERBB2* amplification is a marker of poor prognostic that can be reversed by the administration of HER2 inhibitors [4]. This has been one of the best examples of a biomarker that is both prognostic and predictive of treatment response. Trastuzumab has also demonstrated an OS benefit in oesogastric and colorectal adenocarcinomas [6, 7].

Besides trastuzumab, several HER2-directed agents have been successfully developed in the clinic; lapatinib, a reversible tyrosine kinase inhibitor (TKI) of EGFR and HER2, trastuzumab emtansine (T-DM1), an antibody-drug conjugate, and pertuzumab in association with trastuzumab have also demonstrated an OS benefit in breast cancer [8–10].

In addition to amplification, mutations have been described in *ERBB2* that occur at low frequency in several tumor types, especially in breast (3%) [11], colon (2-3%) [12] and lung cancers (1-2%) [13]. The sensitivity of *ERBB2* hotspot mutations p.S310Y, p.L755S and p.V842I to HER2-directed treatments have been recently investigated [14–17].

Regarding the increasing number of different types of *ERBB2* alterations described across various tumor types, together with the increasing number of HER2-directed therapies, a prospective and systematic evaluation of *ERBB2* alterations and drug sensitivity should help clarifying future personalized treatment decisions. The MOSCATO-01 and 02 programs propose multiple high-throughput genomic analyses on a fresh tumor biopsy to match targeted molecular agents for patients with various types of cancers refractory to conventional treatments [18]. In the MOSCATO-01 study, on the 1036 adult patients included, a molecular analysis has been successfully performed in 844 patients that allowed the administration of a matched targeted therapy in 199 patients. The progression free survival (PFS) with the targeted-therapy (PFS2) was 1.3 times superior to the PFS on the previous treatment line (PFS1) in 33% of patients. Importantly, the highest PFS2/PFS1 ratio in this study has been achieved in the subgroup of patients with *ERBB2* genomic alterations (65%, *n =* 24), leading us to further analyze in depth this molecularly enriched cohort of patients.

The recent approval by the FDA of anti-PD1 immunotherapies for microsatellite instability-High and mismatch repair deficient cancers independently of the tumor types pave the way to broader drug approval for histology-agnostic but biomarker positive patients [19]. A comprehensive evaluation of well-studied biomarkers that lead to treatment approval is required [20]. Programs for the broad evaluation of these strategies, called “umbrella studies”, are ongoing for numerous molecular targeted agents [21]. In this regard, we hypothesized that a refined analysis focused on patients with various types of somatic *ERBB2* alterations detected in MOSCATO-01 and ongoing MOSCATO-02 would help to precise the landscape of drug-target relationship.

## RESULTS

### Patient characteristics for pooled MOSCATO-01 and 02 with ERBB2 alterations

From the beginning of MOSCATO-01 in December 2011 until January 2017, 1036 patients were included, and after 8 months of accrual in the MOSCATO-02, 262 more patients were included. On these pooled cohorts of patients 934 had a successful molecular portrait of their tumor (759 CGH and 912 tNGS). A new alteration in the *ERBB2* gene have been found in 4.7% (*n =* 44/934) of patients including 1.5% (*n =* 14/912) *ERBB2* mutations (pathogenic variant), and 4% (*n =* 30/759) *ERBB2* amplifications. These patients were affected 13 different tumor types, and had previously received a median of 3 treatments lines (Table 1).

**Table 1:** Patient characteristics

	All patients (*N* = 44)	Evaluable patients (*N* = 31)
Age at inclusion		
Median (range)	56 (20–77)	57 (30–77)
Sex		
Male	24 (55%)	17 (55%)
Female	20 (45%)	14 (45%)
ECOG performance status		
0	14 (32%)	12 (39%)
1	28 (64%)	18 (58%)
2	2 (4%)	1 (3%)
Tumor type		
Head and neck	2 (4.5%)	2 (6.5%)
Colon	3 (7%)	1 (3.2%)
Lung	6 (13.5%)	4 (13%)
Biliary tract cancers	7 (16%)	5 (16%)
Pancreas	1 (2%)	0
Oesogastric	9 (20.5%)	8 (26%)
Breast	7 (16%)	5 (16%)
Cervix	2 (4.5%)	2 (6.5%)
Endometrial	1 (2%)	1 (3%)
Urological	3 (7%)	0
Salivary glands	2 (4.5%)	2 (6.5%)
Neuroendocrine	1 (2%)	1 (3.2%)
Number of metastatic sites		
Median (range)	2 (1–4)	2 (1–4)
Number of previous therapies for advanced disease		
Median (range)	3 (0–11)	3 (0–8)

**Figure 1:**
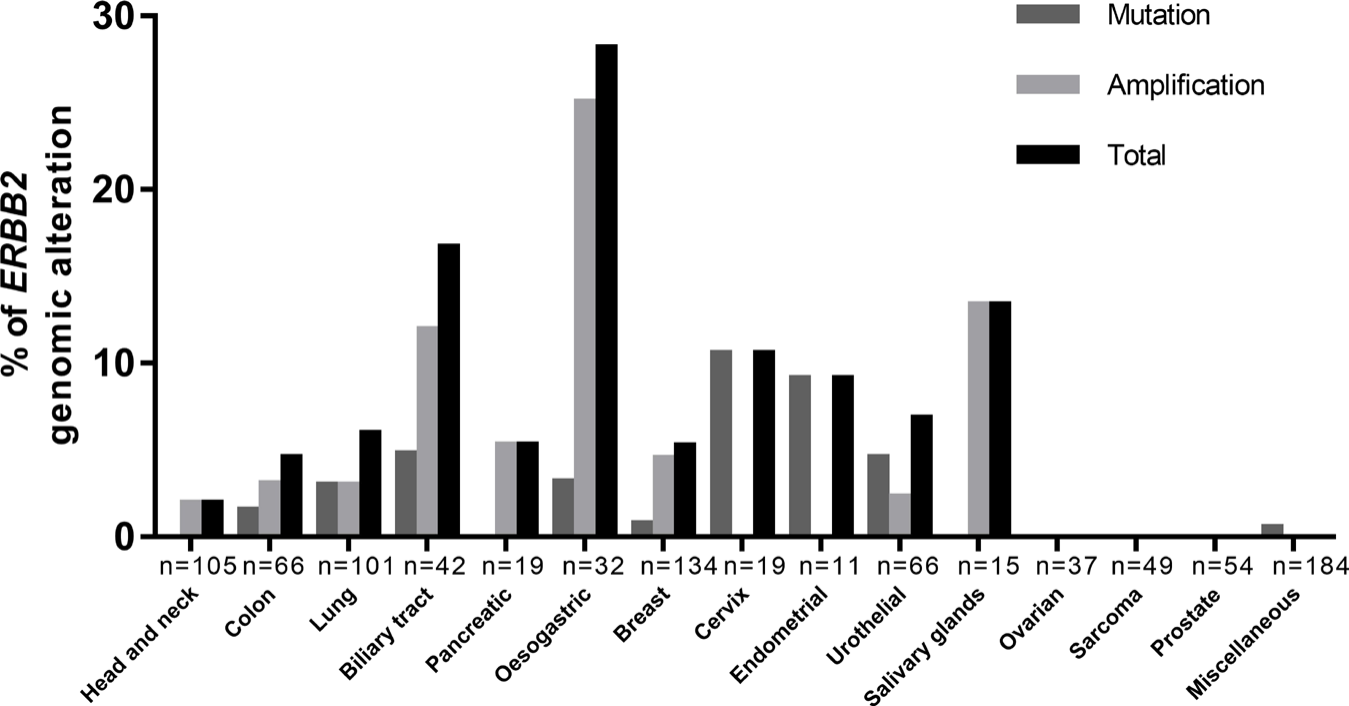
Distribution of ERBB2 genomic alterations, mutation or amplification, among cancers ERBB2 amplifications were mostly found in: salivary gland carcinoma (13%), biliary tract cancers (12%), pancreatic adenocarcinomas (5.3%), lung cancers (3%), with exclusion of breast or oesogastric adenocarcinoma. ERBB2 mutations were mostly found in: cervix carcinomas (10%), endometrial carcinomas (9%), lung cancers (3%), urothelial carcinomas (4.5%), biliary tract carcinomas (*n =* 4.8%).

The frequency of newly detected *ERBB2* amplifications were 25% in oesogastric adenocarcinoma (*n =* 6/32), 13% in salivary gland carcinoma (parotid) (*n =* 2/15), 12% in biliary tract cancers (*n =* 5/42), 5.3% in pancreatic adenocarcinomas (*n =* 1/19), 4.4% in breast cancers (*n =* 6/134), 3% in NSCLC (*n =* 3/101), 3% in colon cancers (*n =* 2/66), 2.3% in urothelial carcinomas (*n =* 1/44) and 1.9% in carcinomas of the head and neck (*n =* 2/105) (Figure 1). The frequency of *ERBB2* mutations were 10% in cervix carcinomas (*n =* 2/19), 9% endometrial carcinomas (*n =* 1/11), 3% in NSCLC (*n =* 3/101), 4.5% in urothelial carcinomas (*n =* 2/44), 4.8% in biliary tract carcinomas (*n =* 2/42), 3.1% in oesogastric carcinomas (*n =* 1/32), 1.5% in colorectal carcinomas (*n =* 1/66), 0.7% in breast cancers (*n =* 1/134). No *ERBB2* genomic alterations were found in prostate cancers (*n =* 54), ovarian cancers (*n =* 37) or sarcomas (*n =* 49).

The known hotspot mutations in *ERBB2* were also found for several patients in our cohort: p.S310Y (*n =* 6), p.L755S (*n =* 2) and p.V842I (*n =* 2) (Figure 2A). *ERBB2* genomic alterations were frequently associated with *TP53* mutations (58%, *n =* 25/43), and/or other activating or inactivating mutations in the PI3K/AKT/mTOR or the MAPK kinase pathways (26%, *n =* 11/43, Figure 2B). Interestingly, 4.8 % (*n =* 6/128) of patient with a metastatic breast cancer previously diagnosed as HER2-negative were found HER2-amplified in CGH, every case confirmed by immunohistochemistry, which led to the reclassification of these metastatic tumors.

**Figure 2A:**
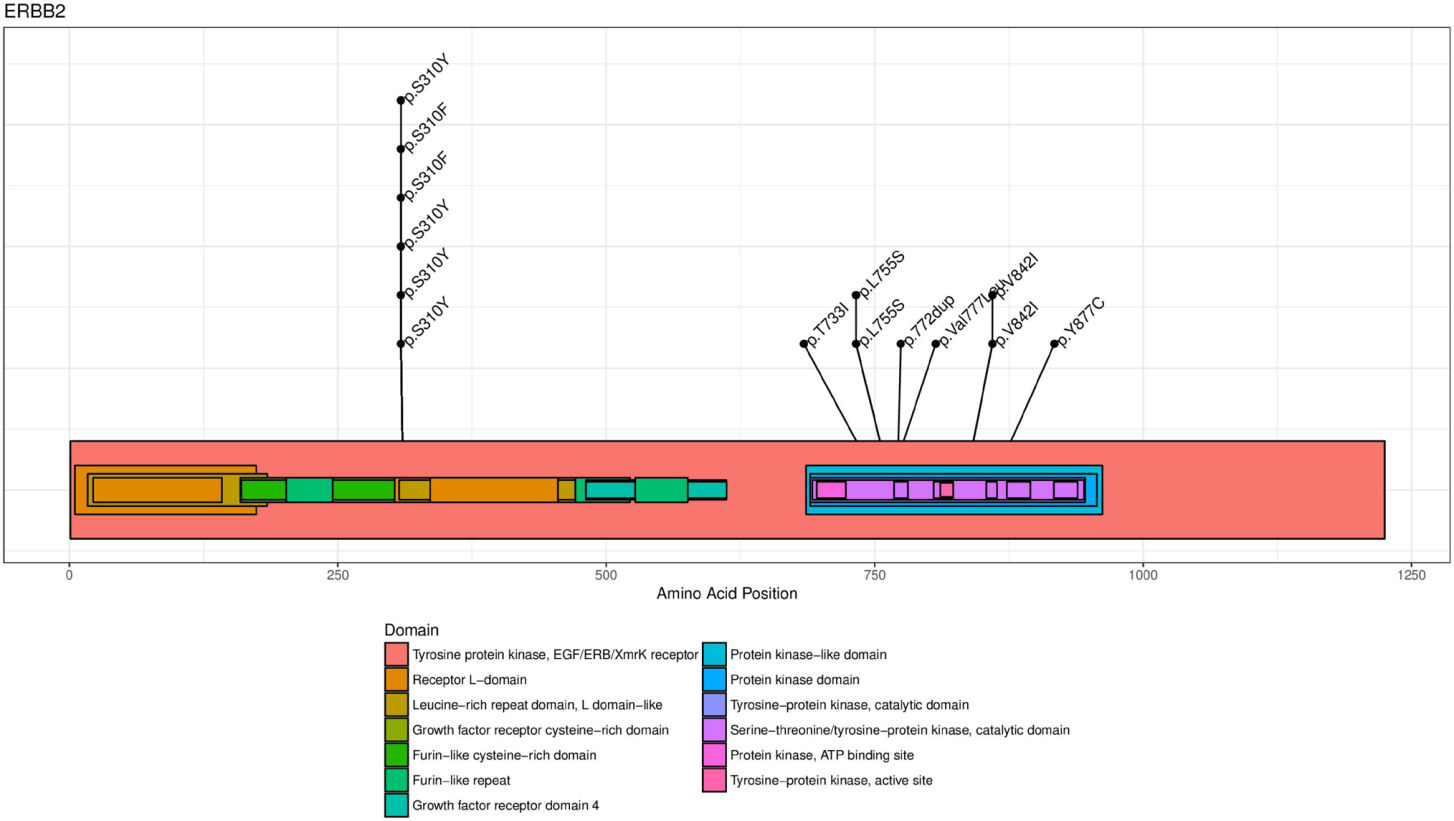
ERBB2 mutation mapping for drivers mutations (*n =* 14) The HER2 protein is represented with known functional sites mapped with different colors reported in the figure legend. The most recurrent hotspot mutations in ERBB2 were p.S310Y (*n =* 6), and the tyrosine kinase domain mutations p.L755S (*n =* 2) and p.V842I (*n =* 2).

**Figure 2B:**
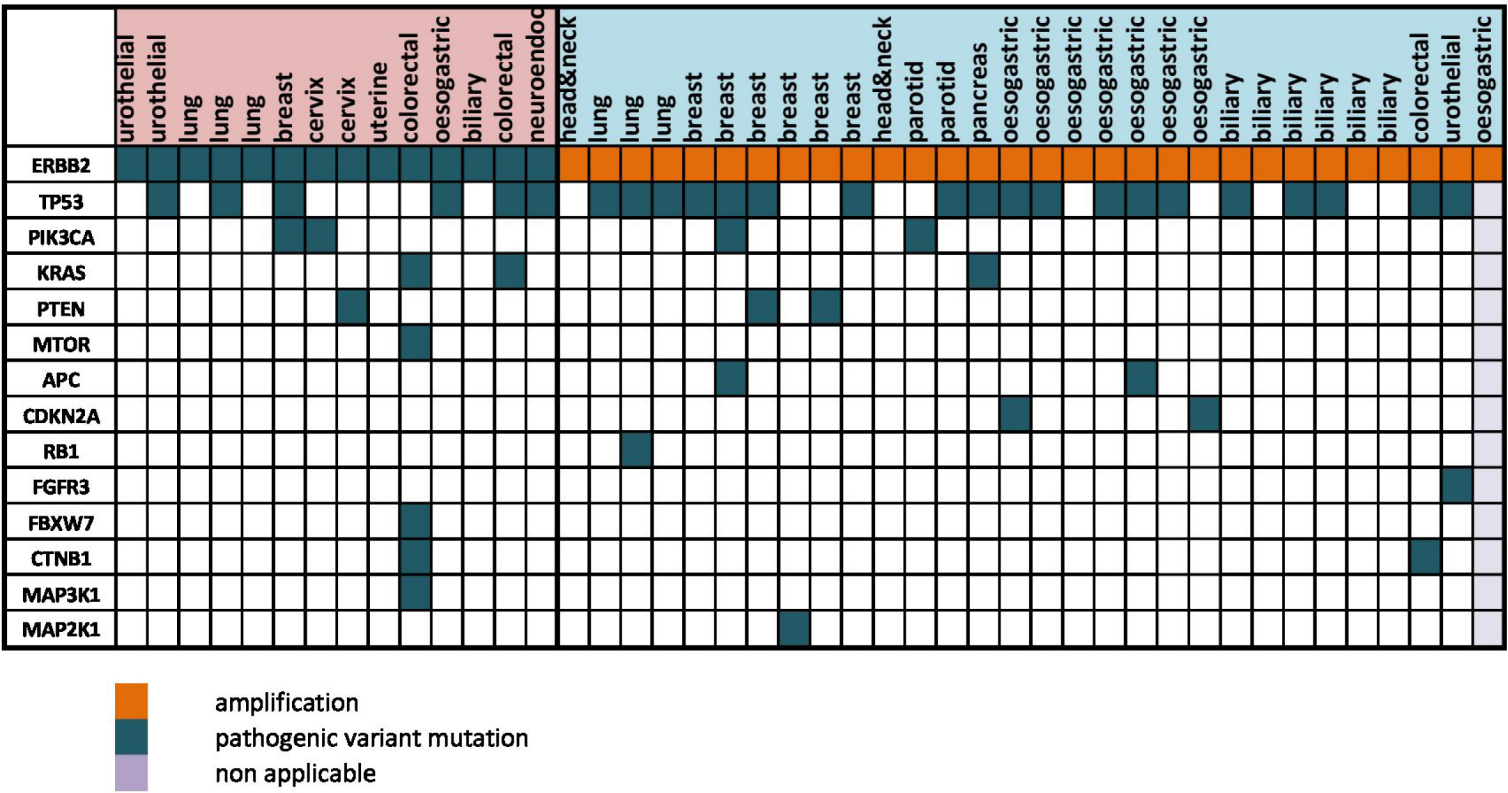
Co-occurrence between ERBB2 mutation/amplification and other mutations Waterfall representation of the altered genes identified in targeted NGS and CGHa, organized relatively to the tumor types. The type of genomic alteration and its functional impact are reported with colors. ERBB2 genomic alterations were frequently associated with TP53 mutations (58%), and/or other activating or inactivating mutations in the PI3K/AKT/mTOR or the MAPK kinase pathways (26%).

### Efficacy of anti-HER2 targeted therapies

On the 31 evaluable patients, 22 were treated with a combination of trastuzumab and chemotherapy (mostly paclitaxel *n =* 13), 6 patients with paclitaxel, trastuzumab and everolimus (due to concomitant mutations in PiK3CA/mTOR pathway), one with neratinib and 2 patients with a combination of trastuzumab and lapatinib. Five patients had breast cancers, 7 oesogastric adenocarcinomas and the remaining 19 patients had 8 different tumor types (Table 1). Concerning these 19 patients with a tumor type other than breast or oesogastric adenocarcinoma, 8 patients had an *ERBB2* mutation and 11 had *ERBB2* amplification.

**Figure 3:**
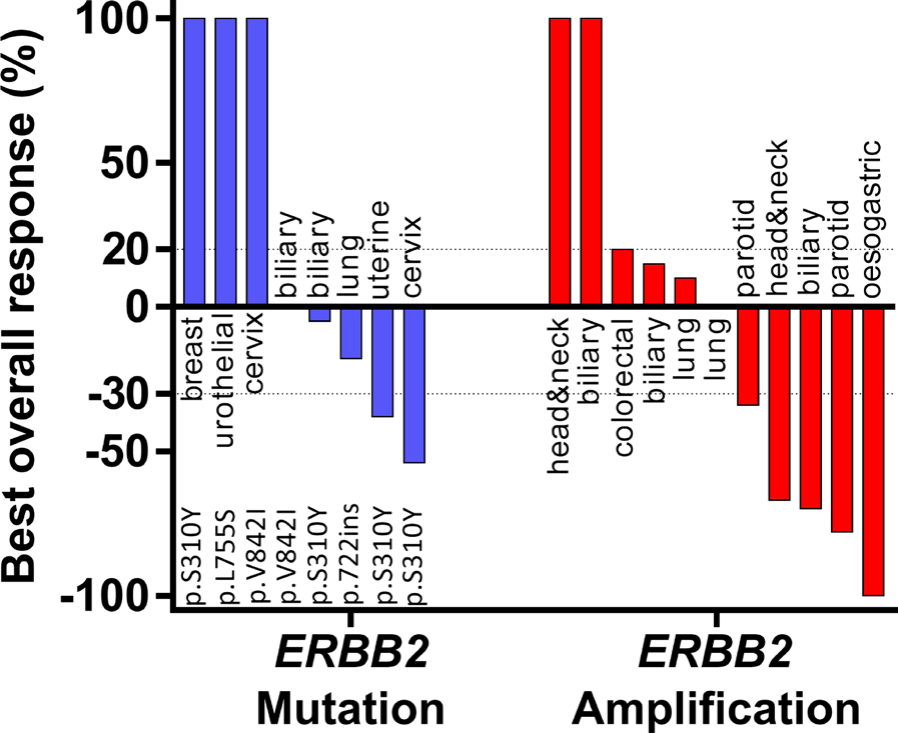
Best overall response rate during anti-HER2 therapy with exclusion of breast or oesogastric adenocarcinoma Best overall response rate was the greater disease in tumor volume under treatment assessed with RECISTS1.1. Patients with early clinical deterioration were arbitrarily put at the maximum observed increase.

**Figure 4:**
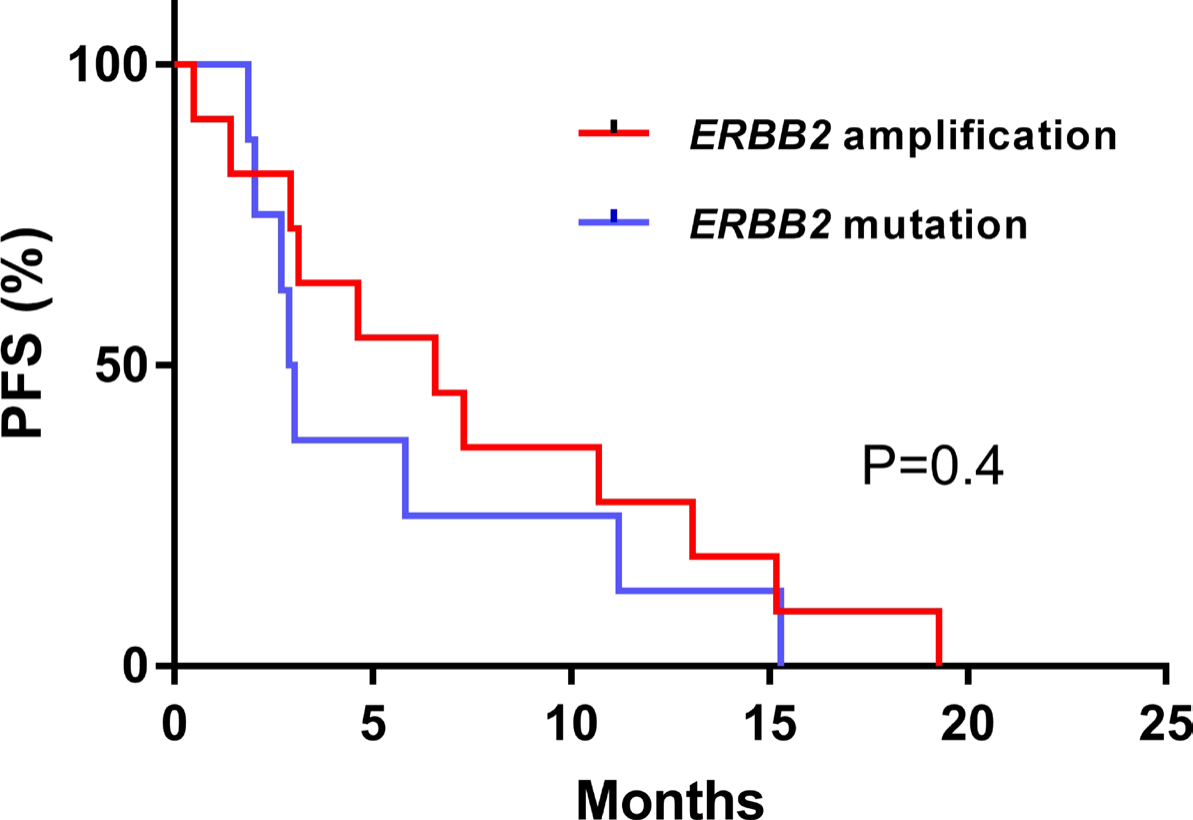
Progression free survival (PFS) according to ERBB2 mutation or amplification therapy with exclusion of breast or oesogastric adenocarcinoma Survival curves were computed with Kaplan Meyer estimation and compared with an unstratified log-rank test.

For the whole cohort of 31 patients, the CBR was 42% (*n =* 13/31, CI95% [25–61%], with 1 CR, 10 PR and 2 SD). For the 19 patients with tumor types other than breast or oesogastric adenocarcinoma, the CBR was 25% (*n =* 2/8 with 2 PR) for *ERBB2* mutation and 64 % (*n =* 7/11 with1 CR, 4 PR, 2 SD > 24 weeks) for *ERBB2* amplification. The two patients who had a PR in the *ERBB2* mutation subgroup received a combination of paclitaxel, trastuzumab and everolimus for an adenocarcinoma of cervix and an endometrial carcinoma, both harboring *ERBB2* S310Y mutation. For somatic *ERBB2* amplification, all patients that achieved CR or PR were treated with chemotherapy plus trastuzumab (only one received paclitaxel, trastuzumab plus everolimus) (Figure 3). For the 19 patients with a tumor type other than breast or oesogastric adenocarcinoma, the median PFS was 4.6 months CI95% [0.7–8.6]; for patients with *ERRB2* mutations the median PFS was 2.9 months CI95% [2.4–3.3] and for patients with *ERBB2* amplification the median PFS was 6.5 months CI95% [2–11], without statistical difference in PFS relative to amplifications or mutations (*p =* 0.4, Figure 4).

## DISCUSSION

This study has demonstrated that *ERBB2* genomic alterations can be found beyond oesogastric and breast cancers in a wide variety of tumor types at low to moderate frequency (between <1% and 13%). The molecular profiling of patients with refractory metastatic tumors allowed identifying non previously detected *ERBB2* amplifications in 6 out of 134 patients with breast cancers [22, 23]. We have also confirmed the high frequency of *ERBB2* amplifications for 13% of patients with salivary gland carcinoma and 5.2% of patients with biliary tract cancers [24].

ERBB2 genomic alterations are supposed to be driver mutations, but patients from our cohort did not receive anti-HER2 therapies prior to the molecular analysis neither performed prior molecular analysis. Therefore, we could not affirm that these ERBB2 alterations were founder or acquired events.

Targeting HER2 in altered tumors led to a CBR of 42% (*n =* 13/31, CI95% [25–61%]), and this benefit was particularly pronounced for *ERBB2* amplifications with a CBR of 64%. These results suggest a strong oncogene addiction to *ERBB2* alterations. In patients with breast and oesogastric adenocarcinoma, results of HER2-directed therapy were consistent with other studies [1, 6]. Trastuzumab plus chemotherapy, mainly paclitaxel, was efficient in patients with *ERBB2*-amplified tumors, as previously described in breast, colon, oesogastric adenocarcinoma [1, 6, 7] and biliary tract cancer [24]. In lung cancer, recent studies showed that a subset of NSCLC is HER2 driven and suggests potential opportunity for HER2 inhibitors in monotherapy or in combination with chemotherapy [13, 14]. Moreover in bladder cancer and colon cancer, several studies have showed promising results of HER inhibition in advanced cancer patients [7, 17, 25].

As patients in the MOSCATO trial were highly pre-treated, the activity of HER2 directed agents may have had a major contribution in the responses even in case of treatment combinations with chemotherapy. We have observed responses for patients with tumor types other than oesogastric or breast and *ERBB2* amplifications. However, we cannot definitely conclude whether the clinical benefit observed in patients was secondary to anti-HER2 directed therapy, chemotherapy or the combination of both. Recently at AACR-NCI-EORTC meeting 2017, E.Ileana *et al.* found *ERBB2* amplifications in 4–14% of various cancer types [26]. In their study they could also confirm that anti-HER2 therapies conferred clinical benefit to patients with tumors beyond classical recommendations.

We found in our study that patients with *ERBB2* mutations had a lower CBR than patients with *ERBB2* amplification, suggesting an influence of the type of alteration on anti-HER2 therapy. However, we should be cautious before drawing a definitive conclusion, since the treatments were not homogeneous and only two patients were treated with a dual anti-HER2 therapy or an irreversible anti-HER2 TKI such as neratinib. The 2 responders treated by chemotherapy plus trastuzumab had an *ERBB2* S310Y mutated tumor, affecting the extracellular domain of HER2, confirming the preclinical demonstration of sensitivity to trastuzumab [17]. Moreover, *in vitro* and *in vivo* data suggest that *ERBB2*-mutated breast cancer are sensitive to neratinib (irreversible anti-HER2 TKI) [27] and that *ERBB2*-mutated colon cancers are more sensitive to dual HER2 inhibition (TKI + Mab) compared to monotherapy or reversible TKI [16, 17]. In the clinic, various HER2-directed therapies have demonstrated an overall response rate of 51% (*n =* 101) in *ERBB2* mutated metastatic lung cancer [14]. Another phase II study evaluating dacomitinib, an irreversible tyrosine kinase inhibitor of HER2, EGFR and HER4, has achieved partial responses in 3 of 26 patients with tumors harboring *ERBB2* exon 20 mutations [28]. Concerning *ERBB2*-mutated breast cancers, a phase II has reported a CBR of 31% in 22 patients treated with neratinib [15]. Such approaches have been substantiated in preclinical findings where dual therapy or irreversible anti-HER2 TKI have been shown effective in case of *ERBB2* hotspot mutations [16, 17, 27]. These observations support the need to tailor the treatment of patients to the type of alterations found in the *ERBB2* gene. Moreover, recent evidences suggest that HER2 inhibitors may be efficient in broader molecular alterations such as *ERBB3* [25, 29, 30].

Large cohorts of patients, multi histology basket trials or tumor agnostic meta-analysis would be required to clarify the algorithm of treatment decision in case of *ERBB2* and *ERBB3* alterations, based on previous preclinical and clinical data available. Hopefully the increasing size of molecular screening programs for metastatic tumors such as MSKCC IMPACT study [31], the Michigan Center [32] or large screening in lung cancer [33] or MULTIPLI program should help in this direction.

In addition to molecular screening programs, the access to targeted treatment should be reinforced in personalized medicine trials as suggested in SHIVA, SAFIR, MOSCATO and NCI-MATCH trials [18, 34–36]. Furthermore, for relatively frequent alterations across tumors such as *ERBB2*, umbrella or basket designs may be proposed such as in the Acsé programs in France [37]. For example, Hymans and colleagues showed that it is possible to evaluate the efficacy of targeted therapies in an enriched population with a low prevalence molecular alterations such as AKT1 mutations [38], BRAF mutation [21], or NTRK translocation [39].

In conclusion, our data advocate for an enlargement of the screening of *ERBB2* mutations and amplifications beyond breast or oesogastric cancers. Furthers studies are warranted to improve the robustness of the relation between the type of molecular alteration and the clinical effect of the drugs.

## PATIENTS AND METHODS

### Patients included in the study

The MOSCATO (MOlecular Screening for CAncer Treatment Optimisation, NCT01566019) trial is a molecular screening program to personalize the treatments of patients referred to the early drug development department (DITEP) at Gustave Roussy. The first part of the MOSCATO program has been recently published, and patients accrual is continuing ever since in MOSCATO-02 [18]. An on-purpose tumor biopsy was performed and immediately fresh-frozen for targeted Next Generation Sequencing (tNGS) and comparative genomic hybridization array (CGHa) after histological control. General inclusion criteria of MOSCATO relied on a performance status of 0–1, a refractory or incurable tumor, and a tumor location accessible to biopsy. Importantly, patients with a known molecular alteration that already has a recommended targeted treatment in France were excluded from the study. In the current study, this exclusion criterion concerned patients treated with HER2 inhibitors for an *ERBB2* amplified breast cancer or oesogastric cancer. Compared to the primary analysis of the MOSCATO program, our cohort of patients could be treated by HER2 inhibitors in a variable timeframe after the molecular screening and other patients were included in the ongoing MOSCATO02. This observation has limited our ability to perform comparative analysis with other patients included in MOSCATO-01.

### Tumor samples and molecular analysis

Methods for tumor sampling and molecular analysis have been described previously [18]. Briefly, tumor biopsies were fresh frozen, tumor cellularity was evaluated histologically, tumor DNA was extracted using DNeasy tissue kit and Qiamp kit respectively (Qiagen, Hilden Germany) according to manufacturer’s instructions. tNGS, covering 74 critical oncogenes or tumor suppressor genes (TSG), was performed using Ion torrent (Ion Torrent PGM, Life Technologies^®^). Variant calling was performed with Torrent Suite™ software, variantCaller (v4.x and higher; ThermoFisher Scientific) using GRCh37 (h19) reference. Variants were then annotated using dbsnp (v138) (http://www.ncbi.nlm.nih.gov/SNP), COSMIC (v69), and dbNSFP (V2.1), using SnpSift (v4.0E) and somatic variant were filtered from the germline analysis [40] [41]. CGHa was performed using SurePrint G3 Human aCGH Microarray 4 × 180K, Agilent technologies, Palo alto, CA [42]. The copy number alterations detected with CGHa were classified into 5 categories, namely deletion, loss, neutral, gain and amplification, using the GISTIC algorithm [43]. Amplifications in GISTIC confirmed by a > ×0.7 log2 ratio with a length less than 10 Mb were considered of interest for the current study.

### Annotation of ERBB2 mutations

To annotated the pathogenicity of the *ERBB2* mutations, we have used SnpEff (v4.0E) and the cbioportal annotation tool [44]. Only pathogenic variants detected in the tumor were selected to orientate the patients to HER2 inhibitors. We have mapped the mutations on a schematic structure of the HER2 membranous receptor using the GenVisR package [45].

### Treatments

Patients harboring a druggable molecular alteration were prospectively oriented and treated in either a phase 1 or an off label use of molecular targeted agent, based on the decision of a molecular tumor board.

### Statistics

Progression free survivals (PFS) were calculated from the first administration of treatment to the date of progressive disease or death. Progressive diseases and response rates were reported according to the RECIST1.1 criteria. Clinical benefit rate (CBR), defined by partial response (PR) or complete response (CR) or stable disease (SD) > 24 weeks. Survival curves were compared with the use of an unstratified log-rank test. The subgroup of patients with *ERBB2* alteration in tumor other than breast cancer or an oesogastric adenocarcinoma was also analyzed independently.

## References

[1] Slamon DJ, Leyland-Jones B, Shak S, Fuchs H, Paton V, Bajamonde A, Fleming T, Eiermann W, Wolter J, Pegram M, Baselga J, Norton L (2001). Use of chemotherapy plus a monoclonal antibody against HER2 for metastatic breast cancer that overexpresses HER2. N Engl J Med.

[2] Cardoso F, Costa A, Senkus E, Aapro M, André F, Barrios CH, Bergh J, Bhattacharyya G, Biganzoli L, Cardoso MJ, Carey L, Corneliussen-James D, Curigliano G (2017). 3rd ESO–ESMO International Consensus Guidelines for Advanced Breast Cancer (ABC 3). Breast.

[3] Smyth EC, Verheij M, Allum W, Cunningham D, Cervantes A, Arnold D, ESMO Guidelines Committee (2016). Gastric cancer: ESMO Clinical Practice Guidelines for diagnosis, treatment and follow-up. Ann Oncol.

[4] Slamon DJ, Clark GM, Wong SG, Levin WJ, Ullrich A, McGuire WL (1987). Human breast cancer: correlation of relapse and survival with amplification of the HER-2/neu oncogene. Science.

[5] Van Cutsem E, Bang YJ, Feng-Yi F, Xu JM, Lee KW, Jiao SC, Chong JL, López-Sanchez RI, Price T, Gladkov O, Stoss O, Hill J, Ng V (2015). HER2 screening data from ToGA: targeting HER2 in gastric and gastroesophageal junction cancer. Gastric Cancer.

[6] Bang YJ, Van Cutsem E, Feyereislova A, Chung HC, Shen L, Sawaki A, Lordick F, Ohtsu A, Omuro Y, Satoh T, Aprile G, Kulikov E, Hill J, ToGA Trial Investigators (2010). Trastuzumab in combination with chemotherapy versus chemotherapy alone for treatment of HER2-positive advanced gastric or gastro-oesophageal junction cancer (ToGA): a phase 3, open-label, randomised controlled trial. Lancet.

[7] Sartore-Bianchi A, Trusolino L, Martino C, Bencardino K, Lonardi S, Bergamo F, Zagonel V, Leone F, Depetris I, Martinelli E, Troiani T, Ciardiello F, Racca P (2016). Dual-targeted therapy with trastuzumab and lapatinib in treatment-refractory, KRAS codon 12/13 wild-type, HER2-positive metastatic colorectal cancer (HERACLES): a proof-of-concept, multicentre, open-label, phase 2 trial. Lancet Oncol.

[8] Geyer CE, Forster J, Lindquist D, Chan S, Romieu CG, Pienkowski T, Jagiello-Gruszfeld A, Crown J, Chan A, Kaufman B, Skarlos D, Campone M, Davidson N (2006). Lapatinib plus capecitabine for HER2-positive advanced breast cancer. N Engl J Med.

[9] Baselga J, Cortés J, Kim SB, Im SA, Hegg R, Im YH, Roman L, Pedrini JL, Pienkowski T, Knott A, Clark E, Benyunes MC, Ross G, Swain SM, CLEOPATRA Study Group (2012). Pertuzumab plus trastuzumab plus docetaxel for metastatic breast cancer. N Engl J Med.

[10] Verma S, Miles D, Gianni L, Krop IE, Welslau M, Baselga J, Pegram M, Oh DY, Diéras V, Guardino E, Fang L, Lu MW, Olsen S, Blackwell K, EMILIA Study Group (2012). Trastuzumab emtansine for HER2-positive advanced breast cancer. N Engl J Med.

[11] Petrelli F, Tomasello G, Barni S, Lonati V, Passalacqua R, Ghidini M (2017). Clinical and pathological characterization of HER2 mutations in human breast cancer: a systematic review of the literature. Breast Cancer Res Treat.

[12] Network TC, Cancer Genome Atlas Network (2012). Comprehensive molecular characterization of human colon and rectal cancer. Nature.

[13] Mazières J, Peters S, Lepage B, Cortot AB, Barlesi F, Beau-Faller M, Besse B, Blons H, Mansuet-Lupo A, Urban T, Moro-Sibilot D, Dansin E, Chouaid C (2013). Lung cancer that harbors an HER2 mutation: epidemiologic characteristics and therapeutic perspectives. J Clin Oncol.

[14] Mazières J, Barlesi F, Filleron T, Besse B, Monnet I, Beau-Faller M, Peters S, Dansin E, Früh M, Pless M, Rosell R, Wislez M, Fournel P (2016). Lung cancer patients with HER2 mutations treated with chemotherapy and HER2-targeted drugs: results from the European EUHER2 cohort. Ann Oncol.

[15] Ma CX, Bose R, Gao F, Freedman RA, Telli ML, Kimmick G, Winer E, Naughton M, Goetz MP, Russell C, Tripathy D, Cobleigh M, Forero A (2017). Neratinib Efficacy and Circulating Tumor DNA Detection of HER2 Mutations in HER2 Nonamplified Metastatic Breast Cancer. Clin Cancer Res.

[16] Kloth M, Ruesseler V, Engel C, Koenig K, Peifer M, Mariotti E, Kuenstlinger H, Florin A, Rommerscheidt-Fuss U, Koitzsch U, Wodtke C, Ueckeroth F, Holzapfel S (2016). Activating ERBB2/HER2 mutations indicate susceptibility to pan-HER inhibitors in Lynch and Lynch-like colorectal cancer. Gut.

[17] Kavuri SM, Jain N, Galimi F, Cottino F, Leto SM, Migliardi G, Searleman AC, Shen W, Monsey J, Trusolino L, Jacobs SA, Bertotti A, Bose R (2015). HER2 activating mutations are targets for colorectal cancer treatment. Cancer Discov.

[18] Massard C, Michiels S, Ferté C, Le Deley MC, Lacroix L, Hollebecque A, Verlingue L, Ileana E, Rosellini S, Ammari S, Ngo-Camus M, Bahleda R, Gazzah A (2017). High-Throughput Genomics and Clinical Outcome in Hard-to-Treat Advanced Cancers: Results of the MOSCATO 01 Trial. Cancer Discov.

[19] Nebot-Bral L, Brandao D, Verlingue L, Rouleau E, Caron O, Despras E, El-Dakdouki Y, Champiat S, Aoufouchi S, Leary A, Marabelle A, Malka D, Chaput N (2017). Hypermutated tumours in the era of immunotherapy: The paradigm of personalised medicine. Eur J Cancer.

[20] Lemery S, Keegan P, Pazdur R (2017). First FDA Approval Agnostic of Cancer Site — When a Biomarker Defines the Indication. N Engl J Med.

[21] Hyman DM, Puzanov I, Subbiah V, Faris JE, Chau I, Blay JY, Wolf J, Raje NS, Diamond EL, Hollebecque A, Gervais R, Elez-Fernandez ME, Italiano A (2015). Vemurafenib in Multiple Nonmelanoma Cancers with BRAF V600 Mutations. N Engl J Med.

[22] Aurilio G, Disalvatore D, Pruneri G, Bagnardi V, Viale G, Curigliano G, Adamoli L, Munzone E, Sciandivasci A, De Vita F, Goldhirsch A, Nolè F (2014). A meta-analysis of oestrogen receptor, progesterone receptor and human epidermal growth factor receptor 2 discordance between primary breast cancer and metastases. Eur J Cancer.

[23] Lindström LS, Karlsson E, Wilking UM, Johansson U, Hartman J, Lidbrink EK, Hatschek T, Skoog L, Bergh J (2012). Clinically used breast cancer markers such as estrogen receptor, progesterone receptor, and human epidermal growth factor receptor 2 are unstable throughout tumor progression. J Clin Oncol.

[24] Javle M, Churi C, Kang HC, Shroff R, Janku F, Surapaneni R, Zuo M, Barrera C, Alshamsi H, Krishnan S, Mishra L, Wolff RA, Kaseb AO (2015). HER2/neu-directed therapy for biliary tract cancer. J Hematol Oncol.

[25] Choudhury NJ, Campanile A, Antic T, Yap KL, Fitzpatrick CA, Wade JL, Karrison T, Stadler WM, Nakamura Y, O’Donnell PH (2016). Afatinib Activity in Platinum-Refractory Metastatic Urothelial Carcinoma in Patients With ERBB Alterations. J Clin Oncol Off J Am Soc Clin Oncol.

[26] Dumbrava EI, Balaji K, Raghav K, Javle M, Blum-Murphy M, Sajan B, Kopetz S, Broaddus R, Routbort M, Pant S, Tsimberidou A, Subbiah V, Hong DS (2018). Abstract A167: Targeting HER2 (ERBB2) amplification identified by next-generation sequencing (NGS) in patients with advanced or metastatic solid tumors. Mol Cancer Ther.

[27] Bose R, Kavuri SM, Searleman AC, Shen W, Shen D, Koboldt DC, Monsey J, Goel N, Aronson AB, Li S, Ma CX, Ding L, Mardis ER (2013). Activating HER2 mutations in HER2 gene amplification negative breast cancer. Cancer Discov.

[28] Kris MG, Camidge DR, Giaccone G, Hida T, Li BT, O’Connell J, Taylor I, Zhang H, Arcila ME, Goldberg Z, Jänne PA (2015). Targeting HER2 aberrations as actionable drivers in lung cancers: phase II trial of the pan-HER tyrosine kinase inhibitor dacomitinib in patients with HER2-mutant or amplified tumors. Ann Oncol.

[29] Bidard FC, Ng CK, Cottu P, Piscuoglio S, Escalup L, Sakr RA, Reyal F, Mariani P, Lim R, Wang L, Norton L, Servois V, Sigal B (2015). Response to dual HER2 blockade in a patient with HER3-mutant metastatic breast cancer. Ann Oncol.

[30] Verlingue L, Massard C, Hollebecque A, Alvarez EC, Postel-Vinay S, Angevin E, Armand JP, Aspeslagh S, Varga A, Ratislav B, Gazzah A, Michot JM, Lacroix L (2016). Clinical efficacy of HER3 partners’ inhibitors in ERBB3 mutated cancer patients. Ann Oncol.

[31] Zehir A, Benayed R, Shah RH, Syed A, Middha S, Kim HR, Srinivasan P, Gao J, Chakravarty D, Devlin SM, Hellmann MD, Barron DA, Schram AM (2017). Mutational landscape of metastatic cancer revealed from prospective clinical sequencing of 10,000 patients. Nat Med.

[32] Robinson DR, Wu YM, Lonigro RJ, Vats P, Cobain E, Everett J, Cao X, Rabban E, Kumar-Sinha C, Raymond V, Schuetze S, Alva A, Siddiqui J (2017). Integrative clinical genomics of metastatic cancer. Nature.

[33] Barlesi F, Mazieres J, Merlio JP, Debieuvre D, Mosser J, Lena H, Ouafik L, Besse B, Rouquette I, Westeel V, Escande F, Monnet I, Lemoine A, Biomarkers France contributors (2016). Routine molecular profiling of patients with advanced non-small-cell lung cancer: results of a 1-year nationwide programme of the French Cooperative Thoracic Intergroup (IFCT). Lancet.

[34] Andre F, Delaloge S, Soria JC (2011). Biology-driven phase II trials: what is the optimal model for molecular selection?. Clin Oncol.

[35] André F, Bachelot T, Commo F, Campone M, Arnedos M, Dieras V, Lacroix-Triki M, Lacroix L, Cohen P, Gentien D, Adélaide J, Dalenc F, Goncalves A (2014). Comparative genomic hybridisation array and DNA sequencing to direct treatment of metastatic breast cancer: a multicentre, prospective trial (SAFIR01/UNICANCER). Lancet Oncol.

[36] Le Tourneau C, Delord JP, Gonçalves A, Gavoille C, Dubot C, Isambert N, Campone M, Trédan O, Massiani MA, Mauborgne C, Armanet S, Servant N, Bièche I, SHIVA investigators (2015). Molecularly targeted therapy based on tumour molecular profiling versus conventional therapy for advanced cancer (SHIVA): a multicentre, open-label, proof-of-concept, randomised, controlled phase 2 trial. Lancet Oncol.

[37] Buzyn A, Blay JY, Hoog-Labouret N, Jimenez M, Nowak F, Deley MCL, Pérol D, Cailliot C, Raynaud J, Vassal G (2016). Equal access to innovative therapies and precision cancer care. Nat Rev Clin Oncol.

[38] Hyman DM, Smyth LM, Donoghue MT, Westin SN, Bedard PL, Dean EJ, Bando H, El-Khoueiry AB, Pérez-Fidalgo JA, Mita A, Schellens JH, Chang MT, Reichel JB (2017). AKT Inhibition in Solid Tumors With AKT1 Mutations. J Clin Oncol.

[39] Hyman DM, Laetsch TW, Kummar S, DuBois SG, Farago AF, Pappo AS, Demetri GD, El-Deiry WS, Lassen UN, Dowlati A, Brose MS, Boni V, Turpin B (2017). The efficacy of larotrectinib (LOXO-101), a selective tropomyosin receptor kinase (TRK) inhibitor, in adult and pediatric TRK fusion cancers. J Clin Oncol.

[40] Forbes SA, Beare D, Gunasekaran P, Leung K, Bindal N, Boutselakis H, Ding M, Bamford S, Cole C, Ward S, Kok CY, Jia M, De T (2015). COSMIC: exploring the world’s knowledge of somatic mutations in human cancer. Nucleic Acids Res.

[41] Liu X, Jian X, Boerwinkle E (2011). dbNSFP: a lightweight database of human nonsynonymous SNPs and their functional predictions. Hum Mutat.

[42] Lazar V, Suo C, Orear C, van den Oord J, Balogh Z, Guegan J, Job B, Meurice G, Ripoche H, Calza S, Hasmats J, Lundeberg J, Lacroix L (2013). Integrated molecular portrait of non-small cell lung cancers. BMC Med Genomics.

[43] Mermel CH, Schumacher SE, Hill B, Meyerson ML, Beroukhim R, Getz G (2011). GISTIC2.0 facilitates sensitive and confident localization of the targets of focal somatic copy-number alteration in human cancers. Genome Biol.

[44] Cerami E, Gao J, Dogrusoz U, Gross BE, Sumer SO, Aksoy BA, Jacobsen A, Byrne CJ, Heuer ML, Larsson E, Antipin Y, Reva B, Goldberg AP (2012). The cBio cancer genomics portal: an open platform for exploring multidimensional cancer genomics data. Cancer Discov.

[45] Durinck S, Spellman PT, Birney E, Huber W (2009). Mapping identifiers for the integration of genomic datasets with the R/Bioconductor package biomaRt. Nat Protoc.

